# The Hypervelocity Impact Behavior and Energy Absorption Evaluation of Fabric

**DOI:** 10.3390/polym15061547

**Published:** 2023-03-21

**Authors:** Huadong Xu, Dong Yu, Jiaxin Cui, Zhixin Shi, Di Song, Changqing Miao

**Affiliations:** 1 National Key Laboratory of Science and Technology on Advanced Composites in Special Environment, Harbin Institute of Technology, Harbin 150001, China; 2 School of Mechanical and Electrical Engineering, Harbin Institute of Technology, Harbin 150001, China; 3 School of Mechanical and Electrical Engineering, University of Electronic Science and Technology of China, Chengdu 611731, China

**Keywords:** micrometeoroid and space debris, hypervelocity impact, shielding performance, fabric, microstructural damage, energy absorption

## Abstract

In this work, the mechanical behavior and energy absorption characteristics of flexible fabric under hypervelocity impact (HVI) were investigated. Basalt fabric, ultra-high molecular weight polyethylene (UHMWPE) fabric, and aluminum alloy (Al) plate were chosen to be the sample materials for their excellent mechanical properties and applicative prospect in spacecraft shielding. HVI experiments had been conducted with the help of a two-stage light-gas gun facility, wherein Al projectile with 3.97 mm diameter was launched at velocities in the range 4.1~4.3 km/s. Impact conditions and areal density were kept constant for all targets. The microstructural damage morphology of fiber post-impact was characterized using a scanning electron microscope (SEM). Analysis results show that a brittle fracture occurred for Basalt fiber during HVI. On the contrary, the ductile fractures with large-scale plastic deformation and apparent thermal softening/melting of the material had happened on the UHMWPE fiber when subjected to a projectile impact. According to the HVI shielding performance and microstructural damage analysis results, it can be inferred that ductile fractures and thermal softening/melting of the material were the prevailing energy absorption behaviors of UHMWPE fabric, which leads to absorbing more impact energy than Basalt fabric and eventually, contributes the superior shielding performance.

## 1. Introduction

High-performance fabrics are often used to resist hypervelocity impact (HVI) in the protection structure of spacecrafts, due to their superior specific strength, specific modulus, and energy-absorbing properties. In addition, the flexible fabrics could be folded and deployed, are conducive to building inflatable structures for use in low earth orbit or lunar bases, and provide protection from micrometeoroid and orbital debris (MMOD) impact. The Stuffed Whipple shield which was used on the on-orbit spacecraft widely incorporates a layer of fabric materials between a bumper and a back metallic plate [1,2,3]. Compared with the metallic material of equal areal density, the intermediate stuffing layer with fabrics could intercept and break the bulk fragments effectively, meanwhile slowing down the velocity and consuming the kinetic energy of the debris cloud. Besides, the rear plate of shielding configuration would not be damaged by the fragments produced by fiber breaking, and the damage of the rear plate would be reduced significantly [4,5,6,7,8].

In the flexible multi-shock shield designed by Christiansen [9,10], multi-layers fabric could consecutively disrupt the projectile to smaller debris and effectively dissipate the shock energy generated by the projectile impact, and improve the shielding efficiency [11,12]. In addition, flexible multi-shock shields could be compressed, folded, and packaged before launch, and then, deploy as inflatable into a several-spaced shield configuration in orbit, thus resolving complications with the extended volume of rigid multi-shock shields [10,13]. Flexible multi-shock shields easily construct large size protective structures on orbit for deployable structures, such as the “TransHab” inflatable module [10], a Model of a flexible inflatable module RSC “Energia” [14], a Bigelow Expandable Activity Module [15].

Polymer and ceramic fabric were used on the spacecraft shielding configuration widely, such as Aramid, ultra-high molecular weight polyethylene (UHMWPE), Basalt, and SiC fabric. Besides the fiber material types, weaving patterns and material properties are also important factors in HVI response and shielding performance. Rudolph et al. [13] conducted HVI tests on different flexible materials and found that the most efficient projectile fragmentation and cloud dispersion were observed on the Kevlar fabric and Refrex fabric target. Cha et al. [16] found that UHMWPE fabric ballistic performance at high temperatures environment was slightly reduced, but in Whipple shield design, the ballistic performance was better than Kevlar. Zhao et al. [17] found through numerical simulation that plain weave fabric had the optimal space debris shielding performance compared with other weave types. The fabric pulls out phenomenon is an important energy absorption mechanism when fabric is impacted by projectiles. Moon et al. [18,19] proposed a new hybrid composite shielding based on a yarn pull-out energy absorption mechanism, for increasing the specific energy absorbing rate. The adhesion degree of Basalt and aramid fabric influences their shielding performance. The over adhesion restricts the fabric layer to absorb the kinetic energy of the debris cloud which causes the shielding performance to degrade [6,7]. Shear Thickening Fluid (STF) impregnated fabric composites [20] and directly curable composites [21] can be more effectively used as a bumper of a hypervelocity shield than as pure fabric layers.

The above works on the HVI characteristics of fabric are mainly focused on the structure design and evaluation of shielding performance. Nonetheless, there is relatively little research conducted on microstructure damage and the energy absorption behavior of fiber under HVI. Moreover, a woven yarn in the fabric is a collection of a bundle of fiber that has a diameter of only several micrometers, and the fabric properties strongly depend on fiber properties and microstructure. To better understand the fundamental energy absorption mechanisms during impact, microstructure damage of fiber materials has been investigated in this work in detail.

It is confirmed from previous investigations [8,16] that Basalt and UHMWPE fabrics have a favorable application prospect in spacecraft shielding due to the excellent mechanical properties and shielding performance. In this work, the HVI experiments of the Basalt fabric, UHMWPE fabric, and 5A06 aluminum alloy (Al) plate, were carried out by using a two-stage light gas gun. Specially, the 5A06 Al plate is selected for comparison of shielding performance, for this material is commonly used to manufacture spacecraft bulkheads [22]. The projectile is a 2017-T4 Al sphere with a diameter of 3.97 mm toward replacing irregular space debris, and the impact velocity range is 4.1~4.3 km/s. Shielding performance was evaluated in terms of energy absorption performance and damage status of witness plate, and the fracture modes and thermal effects of Basalt and UHMWPE fiber were analyzed by utilizing scanning electron microscopy (SEM). The investigation provides useful insights into the energy absorption behaviors of fiber material under HVI.

## 2. Material and Hypervelocity Impact Experiment

HVI experiments were performed on Basalt and UHMWPE fabrics, as well as Al plate. The properties of materials used in this work are described in Section 2.1. The shield configuration and targets used to conduct the HVI experiments are outlined in Section 2.2 and Section 2.3.

### 2.1. Materials

**Figure 1:**
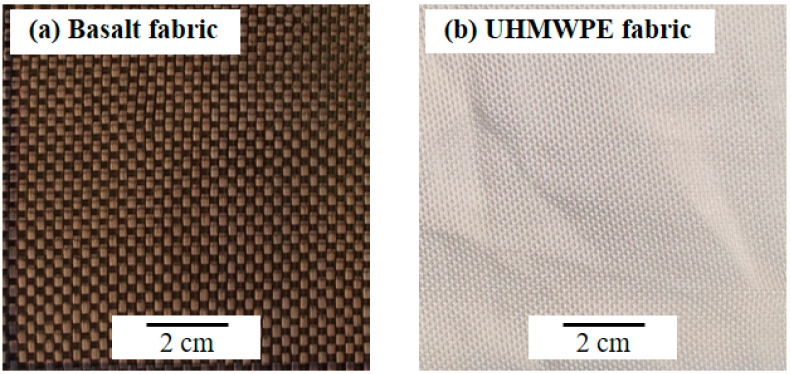
Basalt and UHMWPE fabrics were used in experiments. (**a**) Basalt fabric. (**b**) UHMWPE fabric.

In this study, UHMWPE and Basalt plain woven fabrics were used as experimental materials. Meanwhile, the 5A06 Al plate is selected for comparison of shielding performance. Basalt and UHMWPE plain woven fabrics used in experiments are shown in Figure 1.

Table 1 shows the material properties of the Basalt and UHMWPE fabrics as well as the 5A06 aluminum alloy. The values are obtained from material performance tests or data sheets provided by the manufacturer.

In order to compare the impact resistance of fabric targets and Al plate, the areal densities of all targets were made as close to each other for comparison. Considering the application background of orbital debris shielding, the areal density is an important factor in evaluating the performance of the structures, as the highest performance per areal density can be used as a comparison reference unit for various systems. As the application of fabrics to hypervelocity impact cases intends to take advantage of the flexibility and mobility that such a configuration provides, the performance comparisons were conducted with an interest in the areal density.

From Table 1, the tensile strength of Basalt fiber is approximately 1.5 times that of UHMWPE, but breaking elongation is only about 0.8 times. Young’s modulus for Basalt fiber is approximately consistent with UHMWPE. The thermal properties of the two fabric materials are significantly different. The melting temperature for Basalt and UHMWPE was 1050 °C and 120 °C, respectively. UHMWPE fiber is more likely to soften and melt under the same heating process. Knowledge of the melting temperature can be used to infer the temperatures experienced in materials by post-HVI forensic analysis.

UHMWPE fiber is made up of extremely high molecular weight and long polymeric chains of polyethylene (monomer unit > 250,000 per molecule) aligned in the same direction. The molecular chain rotates through the C-C bond to form a folding chain, forming a regular structure locally, resulting in a highly crystalline and oriented chain of molecules along the fiber direction [23,24]. The degree of crystallinity of commercial fiber varies between 70% and 85%. Such a crystal structure enables UHMWPE fiber to offer a combination of low density, high strength, and high stiffness [25,26]. Basalt fiber is a novel fiber that has appeared in recent years. The average diameter of Basalt fiber was 13 μm. They have high chemical stability and mechanical properties and could be widely applied to many fields. Basalt fiber were previously investigated in shielding configurations [8]. The whole Basalt fiber existed in an amorphous form and remained in a metastable state [27]. Basalt fiber is composed of 52.73 wt% SiO_2_, 15.15 wt% Al_2_O_3_, 5.93 wt% FeO, 4.17 wt% Fe_2_O_3_, 8.61 wt% CaO, 6.37 wt% MgO, and 7.04 wt% Other components [28]. Chemical composition of 5A06 Al are 0.032 wt% Cu, 0.258 wt% Fe, 6.42 wt% Mg, 0.555 wt% Mn, 0.422 wt% Si, 0.0495 wt% Ti, 0.0331 wt% V, 0.0216 wt% Zn, and residual components is Al [29].

### 2.2. Experimental Configuration

**Figure 2:**
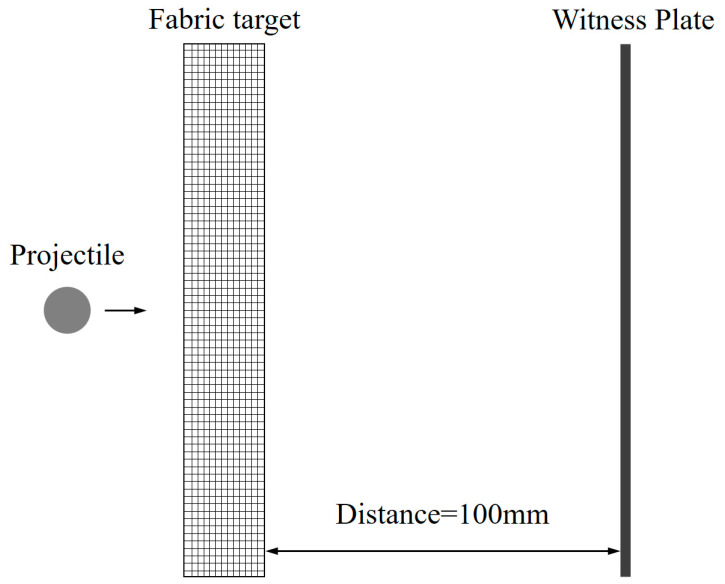
Configuration of the experimental setup.

The schematic of the single shield of fabric for HVI experiments is shown in Figure 2.

The fabric target is made of multi-layers fabric, based on the areal density of a 4 mm thick 5A06 Al plate. All targets have a similar areal density (AD) of about 10,800 ± 200 g/m^3^, with a planar dimension of 200 mm × 200 mm. The detailed parameters of the fabric target, such as the areal density of single layer fabric, the number of layers, the total areal density, and the thickness are shown in Table 1.

The witness plate is installed behind the fabric target, with an approximately 100 mm stand-off distance. The witness plate is composed of 4 layers of 1 mm thickness and 200 mm × 200 mm size 5A06 Al plate, which is convenient to analyze the penetration of the target and the characteristics of fragments generated by the projectile impact. All targets were impacted with 3.97 mm 2017-T4 Al sphere spheres (nominal mass: 0.09 ± 0.002 g) with velocities of 4.1~4.3 km/s.

### 2.3. HVI Experiments

**Figure 3:**
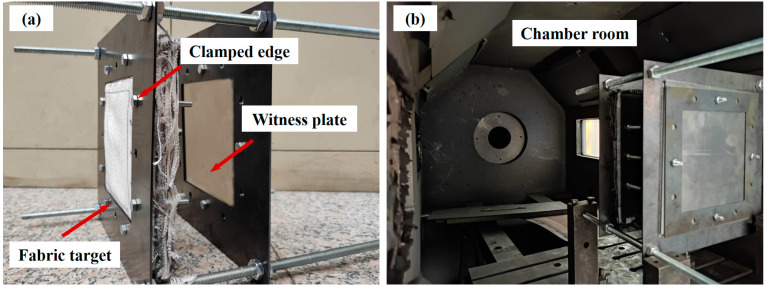
HVI experiments equipment. (**a**) Installation of target. (**b**) Experimental chamber room.

The production and installation process of the UHMWPE fabric target is shown in Figure 3.

The fabric was stacked along the thickness direction and glued on the edge of the fabric, effectively preventing the yarn from pulling off and clamping the edge of the fabric. The target assembly, consisting of the two steel plates and the fabric specimen, was held together by fasteners located around the target, effectively inducing clamped boundary conditions on targets. The target and witness plate are combined by a long bolt to form the single shield structure Figure 3a. A two-stage light–gas gun was used to perform HVI tests in this stud. The installation position of the target in the chamber room is shown in Figure 3b. The chamber room pressure would be lower than 200 Pa when the hypervelocity impact test on shielding configuration was carried out, to provide an approximate vacuum environment for the impact process. The velocity of the projectile was recorded by the laser velocimeter system.

## 3. Results and Discussion

The shielding performance of targets was evaluated in terms of energy absorption performance and damage status of witness plate. Furthermore, the fracture modes and thermal effects of Basalt and UHMWPE fiber were analyzed by the microstructural damage characterization with SEM, to understand the reasons for the difference in energy absorption behaviors and shielding performance.

### 3.1. Shielding Performance Analysis

All the experimental results are summarized in Table 2, which includes measured projectile velocity, target perforation, the volume and maximum depth of the impact crater in the witness plate.

**Figure 4:**
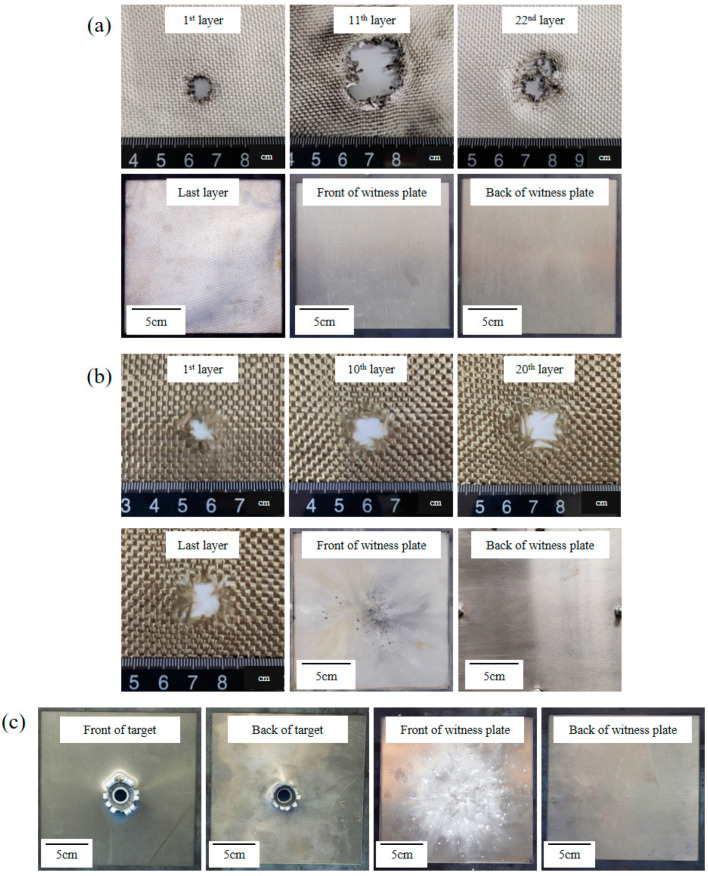
Results of HVI experiment for (**a**) UHMWPE fabric (S3, 4.250 km/s); (**b**) Basalt fabric (S1, 4.190 km/s); (**c**) Al plate (S2, 4.266 km/s).

The results are considerably different, although impact conditions and areal density were kept constant for all targets. Except for UHMWPE fabrics target, other targets were penetrated by projectiles. The impact holes of target and structural damage to witness plate are shown in Figure 4.

The impact results of Basalt fabric target and Al plates are shown in Figure 4b,c, respectively. The Basalt fabric target was penetrated by hypervelocity projectiles. There are several impact craters by projectile debris and fiber debris on the witness plate, which are distributed radially. The back of the witness plate was intact. For the Al plate, the target plate is penetrated by a projectile. There are many impact pits on the front of the witness plate, while the back of the witness plate is intact.

**Figure 5:**
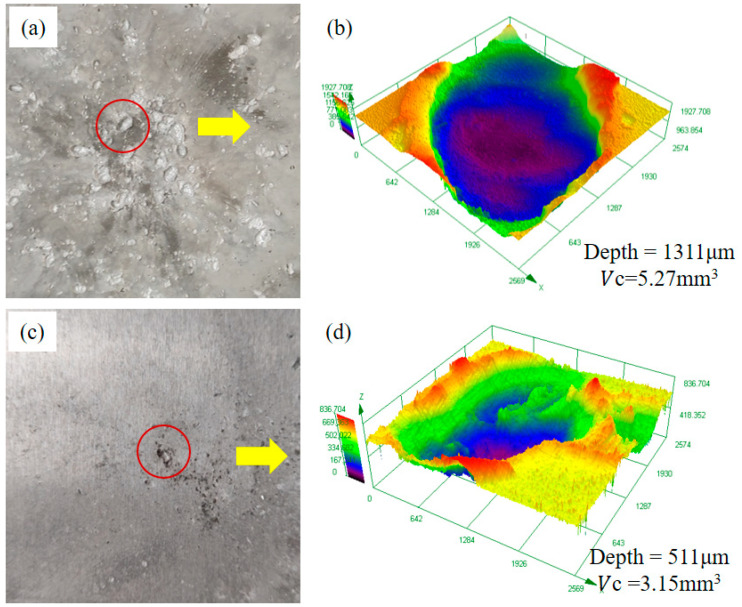
Microscopic morphology of the witness plate. (**a**) Impact crater in witness plate of Al plate. (**b**) Microscopic appearance of impact crater. (**c**) Impact crater in witness plate of Basalt fabric. (**d**) Microscopic appearance of impact crater.

The volume and depth of the impact crater on the witness plate were measured by a 3D measuring laser microscope. The impact crater 3D shape images were shown in Figure 5.

The depth of the impact crater is measured by altitude intercept between the bottom of the impact crater and the non-impact surface. It can be seen from Figure 5b,d that the maximum crater depth of the Basalt fabric and Al plate witness plate were 511 μm and 1311 μm, respectively. It can be inferred that the fragments generated by the projectile impacting the Basalt fabric have smaller kinetic energy and are less destructive. Therefore, compared with the Al plate, the Basalt fabric has a better shielding performance.

**Figure 6:**
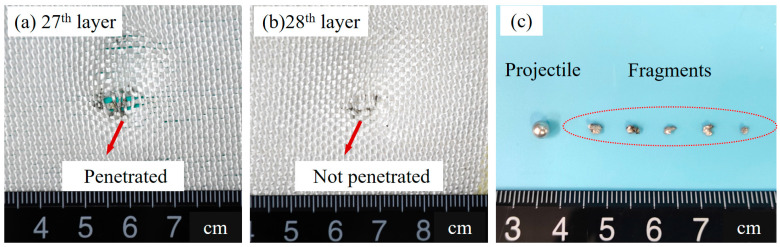
UHMWPE fabrics and projectile fragments. (**a**) 27th layer. (**b**) 28th layer. (**c**) Fragments remaining in fabric.

The HVI experiment results of the UHMWPE fabric target are shown in Figure 4a. The UHMWPE fabric target was not penetrated and the witness plate is intact. The two layers of fabric in the critical state of penetration are shown in Figure 6.

There are 35 layers in the UHMWPE fabric target, of which 27 layers are penetrated by projectiles (Figure 6a), and the remaining 8 layers are not penetrated (Figure 6b). The remaining quality accounts for 22.86% of the total quality. The projectile fragments remaining in the fabric between 27th and 28th are shown in Figure 6c, right side, and their mass is 0.017 g. The left side is the original projectile, with a diameter of 3.97 mm and a mass of 0.090 g.

The specific absorbed energy (SAE) $E_{s}$, which is the absorbed energy divided by the areal density of the penetrated fabric layers, was used to characterize energy absorption performance [30]. It is described as Equation (1):

(1) $$E_{s}=\frac{1}{2}\frac{m_{p}\left(v_{I}^{2}-v_{R}^{2}\right)}{AD_{p}}$$

in which $v_{I}$ and $v_{R}$ represent the velocity of projectile before and after impact, respectively; $m_{p}$ is the projectile mass; $AD_{p}$ is the total areal density of the penetrated fabric layers in target, which is defined as the mass of the total penetrated fabric layers $m_{\mathrm{tp}}$ divided by the area of the impact plane $S_{t}$. Thus, the unit of $E_{s}$ is J/(kg/m^2^).

In experiments S1 and S2, the initial kinetic energies of the projectiles were 790.0 J and 819 J, respectively. The remaining kinetic energy of the projectile and fragments could be calculated by the relationship between the kinetic energy and the volume of the crater [31].

(2) $$V_{c}=713\;E^{1.09}$$

The $V_{c}$ is the volume of impact crater, and *E* is kinetic energy of fragments. It may be noticed that Equation (2) is an empirical formula for Al 2017 projectiles and Al 5056 targets. The chemical composition and material properties of Al 5056 and 5A06 Al plates are similar. Young’s modulus of both materials is 71 GPa, and the tensile strengths are 290 and 339 MPa, respectively. Therefore, it is assumed that Equation (2) is suitable for the current situation.

Without considering the surface scratches of the witness plate, the total volume of all the impact craters is 5.19 mm^3^ and 71.76 mm^3^ for Basalt fabric and Al plate witness plate, respectively. Thus, the remaining kinetic energy of the fragments in experiments S1 and S2 are 11 J and 120 J, respectively. Therefore, the SAE of Basalt and aluminum plates is 71.65 $J\cdot m^{2}\cdot {\mathrm{kg}}^{-1}$ and 64.10 $J\cdot m^{2}\cdot {\mathrm{kg}}^{-1}$.

**Figure 7:**
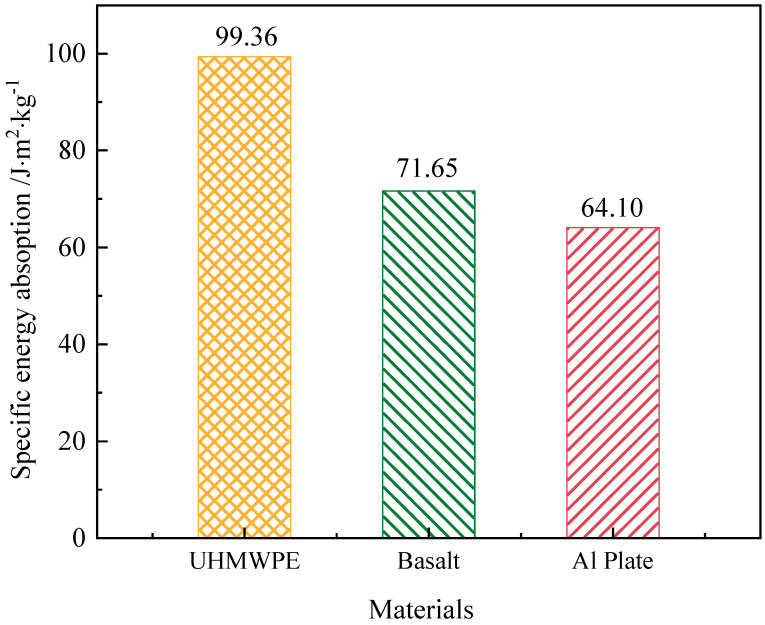
The comparison of specific energy absorption performance.

In experiment S3, the UHMWPE target intercepted the projectile and debris cloud effectively and absorbed all the projectile kinetic energy of 812.8 J. The total areal density of the penetrated fabric layers in target $AD_{p}$ is 8.18 kg/m^2^, Thus, the SAE of UHMWPE target was 99.36 $J\cdot m^{2}\cdot {\mathrm{kg}}^{-1}$. The comparison of the SAE performance of three materials is shown in the Figure 7.

It can be seen from the Figure 7 that the energy absorption efficiency of UHMWPE is greater than that of Basalt and Al plate.

According to the contrast experiments, the UHMWPE fabrics target could be effectively resisting the impact of hypervelocity projectile, while the Basalt fabric target and Al plate were penetrated by the projectile. UHMWPE fabrics exhibited superior energy absorption efficiency and shielding performance than the Basalt fabric and Al plate under the same areal density.

### 3.2. Microstructural Damage Analysis

The fabrics possess a hierarchical multi-scale architecture from fiber to yarns to multi-layer fabrics. Macroscopic response of fabric under HVI strongly depends on the microstructure damage and energy absorption behavior of the fiber. To better understand the fundamental energy absorption mechanisms during impact, microstructure damage of fiber materials, such as fracture modes and thermal effects, requires further investigation.

Preliminary fractography analyses of the fiber damage surfaces using scanning electron microscopy (SEM) reveal distinct differences in fracture modes and energy absorption characteristics in each fiber material.

**Figure 8:**
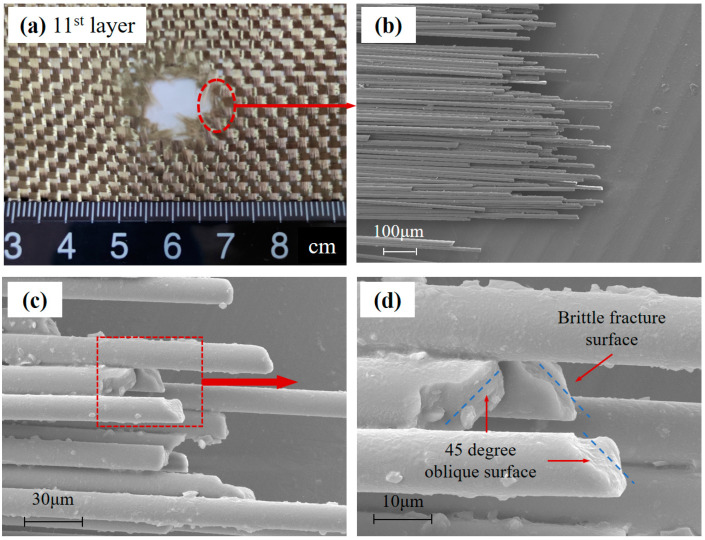
Typical impact feature in Basalt fiber. (**a**) The hole in Basalt fabric. (**b**) Broken Basalt fiber yarn. (**c**) The damaged Basalt fiber. (**d**) Basalt fiber fracture cross-section.

**Figure 9:**
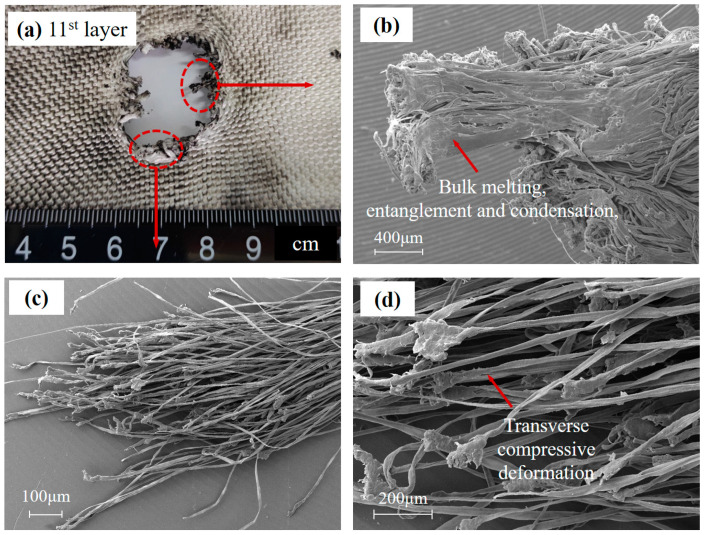
Typical impact feature in UHMWPE yarns. (**a**) The hole in UHMWPE fabric. (**b**) Melting UHMWPE fiber yarn. (**c**) The plastic deformation UHMWPE fiber. (**d**) The broken UHMWPE fiber cross-section.

Typical impact features of Basalt and UHMWPE yarns located at the edge of the hole can be seen in Figure 8 and Figure 9.

In the Figure 8b, the fracture morphology of several Basalt fibers after HVI was approximately consistent. These are exhibited slender cylindrical shapes the same as before impact, and no permanent deformation along the fiber direction. With an increase in the magnifications, the fractographic at fiber-level resolution is shown in Figure 8c,d. The lateral surface of the Basalt fiber exhibits a relatively smooth surface, and a few fiber fragments could be observed scattered on the fiber surfaces. The fracture cross-section for Basalt fiber is fairly rough and wedge-shaped in appearance. The cross-section is oblique with an angle of about 45°, which is the typical brittle fracture pattern. Permanent plastic deformation, such as necking, distortion, and bending, was not found in fracture cross-sections. The fracture morphology characteristics of Basalt fiber accord with the shear failure mode of brittle materials, indicating that brittle fracture occurs during the impact.

Figure 9b,c depict the UHMWPE fiber yarn located at the impact hole of the 22nd layer fabric (Figure 9a). The SEM micrographs show that UHMWPE fiber displays apparent bulk softening and melting of the material and large-scale plastic deformation, such as fibrillation fractures and permanent transverse compressive deformation. Melting fiber at the end of the yarn is entangled and condensed, and eventually, fused (as Figure 9b), possibly as a result of extreme adiabatic heating during impact and lower melting temperature (120 °C) of UHMWPE fiber.

**Figure 10:**
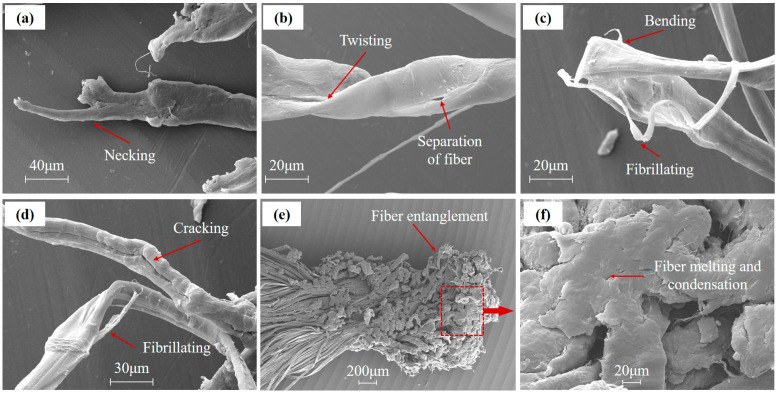
The SEM images of damaged UHMWPE fiber. (**a**) Necking of fiber. (**b**) Twisting and separation of fiber. (**c**) Bending and fibrillating of fiber. (**d**) Cracking of fiber. (**e**) Entanglement of fiber. (**f**) Melting and condensation of fiber.

With an increase in the magnifications, the fracture morphology at fiber-level resolution is shown in Figure 10.

UHMWPE fiber with a nominal fiber of approximately 17 mm is used in this investigation. Different forms of UHMWPE fiber fracture morphology are clearly shown in Figure 10, such as inelastic transverse deformation, necking, twisting damage, and fibrillating. When examining the higher magnification image (Figure 10a–c), the inelastic transverse compression deformation features were observed clearly in the fiber axial. Fractured fiber was flattened into thin slices and separated into fibrils comparing intact fiber, which may be caused by the extreme impact pressure.

When a projectile impacts the fiber transversely and causes large transverse deformation, the fiber can still maintain its load-bearing capability in its longitudinal direction and spread the impact loads. Inelastic transverse deformation of UHMWPE fiber is readily observed from the inspection of the fiber fractographic [32]. UHMWPE fiber can sustain a large amount of transverse compressive strain, likely due to their fibril interactions and the deformation of a mesoscale fibril network [25,33,34]. Transverse compression can be a significant energy absorption mechanism in HVI applications.

In Figure 10a, the fractured fiber shows an obvious necking phenomenon, and the fiber section in necking region decreases gradually, which may be caused by tensile failure. Inter-fibrillar cracks and fibrillating caused by separation of fibrils were observed in Figure 10b–d, and the cracks in fiber surface propagate along the fiber direction. In addition, irreversible plastic deformation such as bending and twisting of fiber was also found in Figure 10b,c. The damage behavior of the fiber discussed above shows that the ductile fracture had happened on UHMWPE fiber during HVI.

The SEM images of the damaged fiber provide valuable insight into the fracture modes and energy absorption mechanism at the fiber level. The crack propagation and fibrillation on the fiber increase the fracture surface area, thereby increasing the energy absorption. In addition, the energy absorbed was further enhanced by irreversible plastic deformation, such as bending, twisting, and transverse compression deformation.

**Figure 11:**
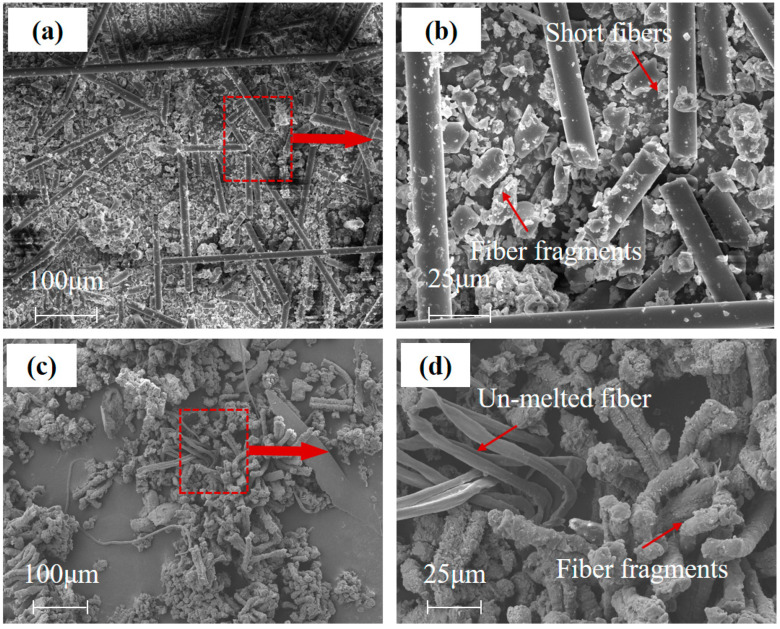
Micromorphology of fiber fragments. (**a**) Fragments of Basalt fiber. (**b**) Broken short fiber and fragments of Basalt fiber. (**c**) Fragments of UHMWPE fiber. (**d**) Un-melted and fragments of UHMWPE fiber.

The Basalt fibers are broken into fragments, due to stronger shock pressures during HVI process. The fiber fragments scattered on the witness plate were sampled and photographed. The SEM images of fiber fragments are shown in Figure 11a,b.

Basalt fiber fragments contain short fiber and fiber powder. The length of Basalt short fiber is about 40~60 μm. Each fiber or fragment did not adhere to each other, and no traces of melting or phase transformation appeared like in UHMWPE fiber. The fragments of UHMWPE fiber are shown in Figure 11c,d. Among the fiber fragments, there are some un-melted and deformed fiber in transverse, and others are rough and obvious melting traces. The melted fiber fragments are bonded together to form a massive fragment.

The comparison results show that Basalt fiber was not subject to a dramatic phase transition during impact as UHMWPE fiber is. It is inferred that the kinetic energy absorbed through phase transformation, such as thermal softening or melting, was little few for Basalt fiber materials. The differences in HVI response for Basalt and UHMWPE fiber material might be depending on the materials’ microstructures (glass network and chain-folded Polyethylene crystals, respectively), chemical composition (aluminosilicate and polymer material, respectively), melt temperature, etc.

## 4. Conclusions

In this study, hypervelocity impact behavior and energy absorption characteristics of Basalt and UHMWPE fabric were analyzed. Basalt and UHMWPE fabrics, and Al plate with equal areal density, were subjected to normal HVI by 3.97 mm diameter Al-2017 aluminum spheres at velocities in the range of 4.1~4.3 km/s. Post-impact analyses of the target specimens were used to provide insight into the microstructural damage and shielding performance of each material, including a 3D laser scanning microscope (LSM), and scanning electron microscope (SEM).

1. Basalt and UHMWPE fiber show different fracture failure modes after HVI with Al projectile. Microstructural damage analysis shows that UHMWPE fiber exhibits ductile fracture and apparent bulk softening/melting of the material, while the Basalt fiber exhibits a typical brittle fracture mode.
2. The UHMWPE fabric target exhibited superior energy absorption efficiency and shielding performance than Basalt fabric and Al plates, while the areal density, projectile size and velocity were similar in the HVI experiments.
3. In summary, the HVI experimental results combined with microstructural damage analysis of fiber materials suggest that ductile fractures with large-scale plastic deformation and apparent thermal softening/melting of fiber are significant energy absorption behavior, which could improve the energy absorption efficiency and the shielding performance.

## Figures and Tables

**Table 1:** Experimental material properties.

Material	Density (g/cm^3^)	Tensile Strength (MPa)	Young’s Modulus (GPa)	Breaking Elongation (%)	Melting Temperature (°C)	1-Layer AD (g/cm^2^)	Number of Layers	Total AD (g/m^2^)	Ref.
Basalt	2.65	3800	93~110	3.1	1050	339.8	32	10,873.6	[8]
5A06	2.64	339	71	25	660	2727.0	4	10,908.0	[22]
UHMWPE	0.96	2470	99	3.7	120	302.8	35	10,598.0	test

**Table 2:** Impact test results.

Experimental	Bumper Material	*V*_proj_/(km/s)	Penetration	Maximum Depth of Impact Crater (mm)	Impact Crater Volume (mm^3^)
S1	Basalt	4.190	Yes	511	5.19
S2	5A06	4.266	Yes	1311	71.76
S3	UHMWPE	4.250	No	0	0

## Data Availability

The raw and processed data generated during this study will be made available upon reasonable request.
